# Evidence concerning parental exposure to pesticides and the occurrence of leukemia in offspring: a systematic review

**DOI:** 10.3389/fped.2025.1560678

**Published:** 2025-04-10

**Authors:** Kennia Cristine de Souza Silva, Geise Ellen Broto Oliveira, Marla Karine Amarante, Carolina Panis

**Affiliations:** ^1^ Programa de Pós-Graduação em Fisiopatologia Clínica e Laboratorial, Universidade Estadual de Londrina, Londrina, Paraná, Brazil; ^2^ Laboratório de Biologia de Tumores, Universidade Estadual do Oeste do Paraná, Campus de Francisco Beltrão, Francisco Beltrão, Brazil

**Keywords:** childhood leukemia, pesticides, parental exposure, transgenerational exposure, risk factors

## Abstract

Leukemias are among the most common childhood cancers. Although its causes are still unclear, parents' environmental exposure to carcinogenic risk factors may have considerable potential. In this context, we revised the literature concerning parental exposure to pesticides, the development of leukemia in offspring, and the underlying molecular mechanisms involved. This systematic review was based on the PRISMA methodology. Only original studies were included; review articles and case reports were excluded. In total, 312 articles were screened. Of the 29 articles selected and 14 were included in this review. The main findings described in the studies above raise the hypothesis that parental pesticide exposure may be related to the development of leukemia in offspring. However, the literature reinforces the lack of well-designed studies highlighting the mechanism triggered by this exposure and its relationship with childhood cancer. The revised literature provides strong evidence supporting the relationship between parental exposure to pesticides and leukemia development in offspring. While gaps remain in understanding the precise mechanisms involved, the findings emphasize the potential risk posed by pesticide exposure and highlight the need for well-designed studies to clarify the underlying biological pathways.

## 1 Introduction

Leukemia ranks 13th among the most common cancers worldwide, with almost 500,000 new cases documented in 2020, remaining a significant public health concern due to its high morbidity and mortality rates across different age groups. Among childhood cancers, leukemia accounts for approximately 30% of all pediatric malignancies, standing out as the main malignant neoplasia (1). It is estimated that most of childhood cancer cases have unknown causes, and emerging evidence suggests that a combination of genetic predisposition and environmental exposures plays a crucial role in leukemogenesis. Identified risk factors are linked to genetic determinants or environmental factors, such as direct or indirect exposure to carcinogenic agents, including ionizing radiation, pesticides, solvents, and viruses (2).

Pesticides are chemical substances extensively utilized in agriculture to control and eliminate pests. However, due to their long half-life and persistent nature, these compounds can infiltrate various environmental compartments, including soil, water, and air, eventually entering the human body through ingestion, inhalation, or dermal absorption (3). Once accumulated, pesticides have been identified as potential contributors to a range of adverse health effects, including endocrine disruption, neurological disorders, and the development of various cancers, including childhood neoplasia, raising significant concerns regarding their long-term impact on human health and well-being, due their multi-scale contamination levels (4).

Parental exposure to pesticides is pointed out as a potential risk factor for leukemia development in the offspring (5). Studies have shown that maternal household and occupational exposure to pesticides increases the risk of childhood leukemia (6, 7). These toxic substances may impact gametogenesis and have the potential to cross the placental barrier, thereby influencing fetal development. Research has demonstrated that pesticide contamination is widespread worldwide, affecting adult individuals, including women. Pesticides have been detected in various biological samples, including breast tissue, breast milk, blood, and urine (8).

Parental exposure to carcinogens during pregnancy may negatively impact fetal development, potentially contributing to leukemogenesis even during intrauterine life (9). Exposure to pesticides can have harmful effects on maternal and fetal health. During pregnancy, the developing fetus is susceptible to pesticide contaminants due to underdeveloped metabolic pathways, accelerated fetal growth, and the formation of essential organ systems. As a result, perinatal exposure to these toxic substances may contribute to unfavorable pregnancy outcomes, posing significant risks to both the mother and child (10).

The mechanisms through which pesticides affect offspring are multifaceted, involving a combination of epigenetic, genetic, and inflammatory changes (11, 12). Environmental exposure to pesticides can induce oxidative stress, resulting in abnormal chromosomal translocations, increased cellular proliferation, and, ultimately, the onset of leukemia (13). Also, double-stranded DNA breaks, which can occur due to exposure to certain mutagenic chemicals, or indirectly through the modulation of type II topoisomerase enzymes, can lead to chromosomal abnormalities that contribute to the development of childhood leukemia (14).

Despite these well-documented mechanisms, there remains limited discussion in the literature about the concrete evidence linking parental pesticide exposure to the development of leukemia in offspring. Considering this, it is necessary to understand how intrauterine exposure impacts subsequent generations and whether pesticides can be considered risk factors for the development of cancer in childhood. While existing studies suggest an association, there is a lack of well-designed research that clarifies the specific biological pathways and causal mechanisms involved in this relationship. Therefore, this review seeks to address the gap in understanding the underlying molecular mechanisms linking parental pesticide exposure to leukemia development in offspring.

## 2 Methods

This study aimed to systematically review the relationship between parental exposure to pesticides and the occurrence of leukemia in offspring. The study is registered in the PROSPERO International Prospective Register of Systematic Reviews under the registration number CRD42024504411.

**Figure 1:**
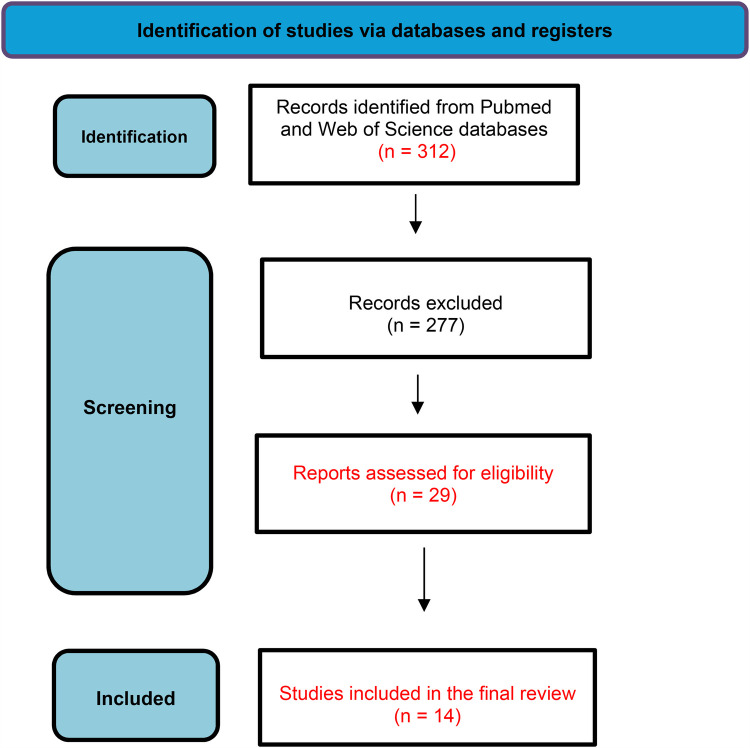
PRISMA flow diagram.

This systematic review was based on the PRISMA 2020 method guidelines (Figure 1). The design of the study is shown in Box 1. The data were obtained from studies available in two critical databases (PubMed—https://www.pubmed.com and Web of Science—https://www.webofscience.com). The search was restricted to articles published from January 2012 to January 2025.

Box 1 **Study design.**

**Table:** 

Information	Criteria
Eligibility criteria	Studies examining parental exposure to pesticides, leukemia, and/or other pediatric tumors were initially screened. To be included, studies had to specifically investigate the association between parental pesticide exposure and the risk or diagnosis of leukemia in offspring. Studies that focused on pediatric tumors other than leukemia or did not directly assess parental pesticide exposure were excluded. Non-peer-reviewed studies were excluded.
Information sources	Literature available at Pubmed database (https://www.pubmed.com) and Web of Science (https://www.webofscience.com) published from January 2012 to March2025.
Search strategy	The mesh terms “parental exposure, pesticides, and leukemia”, and “parental exposure, pesticides, and pediatric tumors” were used. All types of human studies were included. The risk of bias in the studies was assessed using both the Cochrane Risk of Bias tool and the Newcastle-Ottawa Scale (NOS). All studies evaluated were classified as having a low risk of bias.
Selection and data collection process	After applying the search strategy, a total of 312 publications were retrieved. Three independent reviewers red all abstracts and selected the original studies matching the eligibility criteria for full reading (*n* = 29). Information about the type of exposure (domestic, occasional, or parental) were obtained. After this, 14 articles focused on parental exposure were selected for the final systematic review.
Data items	The outcome analyzed was the occurrence of pediatric leukemia in the offspring of parents exposed to pesticides.
Effect measures	When available, risk ratios, odds ratios, confidence intervals, and data percentages were used in the synthesis or presentation of results.
Synthesis methods	A table containing data about study information, country, outcome, and association between parental exposure to pesticides and leukemia occurrence in the offspring is shown (Table 1).

The eligibility criteria initially included studies that examined parental exposure to pesticides, leukemia, and/or other pediatric cancers. To be included, studies had to specifically investigate the relationship between parental pesticide exposure and the risk or diagnosis of leukemia in children. Studies focusing on pediatric cancers other than leukemia or those not directly assessing parental pesticide exposure were excluded, as were non-peer-reviewed articles.

The MeSH terms used were “parental exposure, pesticides, and leukemia” and “parental exposure, pesticides, and pediatric tumors.” All types of human studies were considered. The risk of bias in the studies was assessed using the Cochrane Risk of Bias tool and the Newcastle-Ottawa Scale (NOS), with all studies categorized as having a low risk of bias.

For the initial screening, three authors independently reviewed titles and abstracts to identify eligible articles, with full texts examined when necessary. All included studies were published in English, and only human studies were considered. A total of 312 publications were retrieved after applying the search strategy. Three independent reviewers examined all abstracts and selected 29 studies that met the eligibility criteria for full reading. Data on the type of exposure (domestic, occasional, or parental) were collected. Ultimately, 14 articles focused on parental exposure were selected for the final systematic review.

The primary outcome analyzed was the occurrence of pediatric leukemia in children of parents exposed to pesticides. When available, risk ratios, odds ratios, confidence intervals, and data percentages were used to synthesize and present the results. A table with study information, country of origin, outcome, and the association between parental pesticide exposure and leukemia in offspring is shown (Table 1).

**Table 1:** Characteristics of the selected studies for the systematic review.

Reference	Outcome	Risk Association?
Oniyje et al., 2022 (15)	No associations were observed for paternal exposures to polyaromatic hydrocarbons (OR: 1.00)	No
Patel et al., 2020 (28)	Risk of childhood leukemia was associated with higher crop area near mothers' homes during pregnancy. Compared to mothers living more than 500 meters away from crops, they observed a rising risk of childhood leukemia in offspring as the crop area near the home increased. In the highest exposure category (>24 hectares), the hazard ratio was 2.0 (CI: 1.02–3.8).	Yes
Park et al., 2020 (17)	Elevated risks for ALL with exposure to any carcinogenic pesticide exposure aOR: 2.83, 95% CI: 1.67–4.82), diuron (single-pesticide model, aOR: 2.38, 95% CI: 1.57–3.60), phosmet (OR: 2.10, 95% CI: 1.46–3.02), kresoxim-methyl (OR: 1.77, 95% CI: 1.14–2.75), and propanil (OR: 2.58, 95% CI: 1.44–4.63). Analyses based on chemical classes showed elevated risks for the group of 2,6-dinitroanilines (OR: 2.50, 95% CI: 1.56–3.99), anilides (OR: 2.16, 95% CI: 1.38–3.36), and ureas (OR: 2.18, 95% CI: 1.42–3.34).	Yes
Patel et al., 2020b (18)	Paternal exposures to pesticides were associated with increased risk of childhood AML (herbicides HR = 3.22, 95% CI:0.97–10.68; insecticides HR = 2.86; 95% CI: 0.99–8.23).	Yes
Wang et al., 2019 (19)	Maternal prenatal exposure to pesticides (aOR: 1.48; 95% CI: 1.67–2.28) increased the risk of childhood ALL.	Yes
Hyland et al., 2018 (20)	Self-reported maternal insecticide use inside the home in the year before pregnancy (aOR: 1.63; 95% CI: 1.05–2.53), during pregnancy (aOR: 1.75; 95% CI:1.13–2.73), and while breastfeeding (aOR: 1.75: 95% CI: 1.12–2.73) was associated with increased odds of ALL among boys.	Yes
Ferri et al., 2018 (21)	Increased risk was associated with prenatal maternal use of insecticides/rodenticides (OR:1.87; 95% CI: 1.04–3.33), with subjects living <100 m from pesticide-treated fields (OR: 3.21; 95% CI: 1.37–7.53)	Yes
Gunier et al., 2017 (22)	ALL risk was elevated in young children with paternal occupational to any pesticide exposure during the perinatal period (OR: 1.7; 95% CI: 1.2–2.5). The risk was even higher for exposure to pesticides used on nut crops (OR: 4.5; 95% CI: 0.9–23.0) and for children diagnosed before the age of five (OR: 2.3; 95% CI: 1.3–4.1).	Yes
Bailey et al., 2015 (23)	ALL was associated with any pesticide exposure shortly before conception, during pregnancy and after birth (OR: 1.39; 95% CI: 1.25–1.55) (using 2,785 cases and 3,635 controls), OR: 1.55 (using 2,785 cases and 3,635 controls), OR: 1.43; 95% CI: 1.32, 1.54 (5,055 cases and 7,370 controls) and OR: 1.36; 95% CI: 1.23, 1.51 (4,162 cases and 5,179 controls), respectively. And for AML ORs were were 1.49 (95% CI: 1.02–2.16) (173 cases and 1,789 controls), 1.55 (95% CI: 1.21–1.99) (344 cases and 4,666 controls) and 1.08 (95% CI: 0.76–1.53) (198 cases and 2,655 controls).	Yes
Bailey et al., 2014 (24)	Increased risk of acute myelocytic leukemia (AML) in the offspring with maternal exposure to pesticides during pregnancy. For maternal exposure, the risk of ALL was 1.01 (95% CI 0.78–1.30), while paternal exposure around conception was associated with a slightly increased risk (OR: 1.20, 95% CI: 1.06–1.38). For AML, maternal exposure during pregnancy was linked to a higher risk (OR: 0.91, 95% CI: 0.66–1.24).	Yes
Shi et al., 2013 (25)	Parental exposure to chemicals may increase the risk of childhood acute leukemia in their offspring. Risk factors for childhood acute leukemia may include maternal exposure to various chemicals—such as diesel oil, gasoline, paints, insecticides, pesticides, herbicides, and chemical fertilizers—from three months before pregnancy until its conclusion (OR: 2.9, 95% CI: 1.1–7.8). Additionally, paternal exposure to insecticides (OR: 10.1, 95% CI: 1.2–82.9) and chemical fertilizers (OR: 9.5, 95% CI: 1.1–79.6) within three months before conception was associated with an increased risk. Furthermore, maternal work experience in agriculture and forestry before pregnancy was also linked to a higher risk of childhood acute leukemia (OR: 8.4, 95% CI: 1.4–50.2).	Yes
Ferreira et al., 2013 (26)	Pesticide exposure during pregnancy may be involved in the etiology of acute leukemia in children <2 years of age. Associations with the use of pesticides during pregnancy were found for both ALL (aOR: 2.10; 95% CI: 1.14–3.86) and AML (aOR: 5.01; 95% CI: 1.97–12.7) in children aged 0–11 months, as well as for ALL (aOR: 1.88; 95% CI: 1.05–5.23) in children aged 12–23 months. For maternal exposure to permethrin, higher risk estimates were observed in ALL children–aged 0–11 months (aOR of 2.47; 95% CI: 1.17–5.25), and AML children (OR: 7.28 95% CI: 2.60–20.38). Additionally, maternal pesticide exposure related to agricultural activities was associated with an aOR of 5.25 (95% CI: 1.83–15.08) for ALL and 7.56 (95% CI: 1.83–31.23) for AML.	Yes
Glass et al., 2012 (16)	Paternal occupational exposure to pesticides was not found to be associated with an increased risk of ALL in the offspring for any pesticide exposure (OR: 1.06; 95% CI 0.73–1.55). The study was underpowered with respect to maternal exposure to pesticides.	No
Ruth et al., 2023 (27)	Exposure to termite insecticides during pregnancy was linked to the highest risk for childhood acute lymphoblastic leukemia (adjusted odds ratio: 4.21; 95% confidence interval: 2.00–8.88).	Yes

ALL, acute lymphocytic leukemia; AML, acute myelocytic leukemia; aOR, adjusted odds ratio; CI, confidence interval; OR, odds ratio.

The study used the PECO (Population, Exposure, Comparator, Outcome) strategy as follows:

• P (Population): Children diagnosed with ALL. Studies may include both cases and controls (healthy children or children with other health conditions).
• E (Exposure): Parental pesticide exposure, which may include both maternal and paternal exposure. Exposure could be related to different types of pesticides and could occur during pregnancy, before conception, or during the postnatal period. Exposure may be measured through environmental monitoring, self-reports, or occupational data.
• C (Comparator): Different levels of pesticide exposure. Some studies may include non-exposed parents from the general population.
• (Outcome): Occurrence of ALL in offspring.

## 3 Results

The systematic review analyzed 14 studies investigating the relationship between parental exposure to pesticides and leukemia in offspring. The findings suggest a potential association in 12 studies, with multiple studies indicating an increased risk of leukemia among children whose parents were exposed to pesticides before conception or during pregnancy (Table 1).

Two studies (15, 16) included in this review revealed that there is no close relationship between the appearance of a child having leukemia and parenteral exposure to pesticides.

Onyije et al. (15) studied 3,320 children with leukemia and 6,268 controls; 2,573 of these patients with ALL-B, 340 ALL-T, 376 AML and 73 unknown subtypes. The diagnoses occurred from 1988–2012. The data collected included information from one year before conception (father and mother) and during pregnancy (mother), and mainly concerning the type of work performed during this period. The potentially carcinogenic substances evaluated were polycyclic aromatic hydrocarbons (PAHs), diesel engine gases (DEEs), chromium, nickel, crystalline silica, and asbestos. Odds ratios (ORs) and 95% confidence intervals (CIs) were estimated via unconditional logistic regression models for all childhood leukemias combined with type of leukemia (ALL and AML) and by subtype of acute leukemia (lineage B and T). An association was found between high paternal exposure to crystalline silica and ALL (OR: 2.20, 95% CI 1.60–3.01), with an increasing trend from no exposure to high exposure (*p* =< 0.01) to LMA (OR: 2.03, 95% CI: 1.04–3.97; *P* for trend: 0.008). Similarly, the ORs were similar for B- and T-lineage ALL patients. For ALL patients, the ORs were also slightly elevated, with wide confidence intervals (CIs), for high paternal occupational chromium exposure (OR: 1.23, 95% CI: 0.77–1.96) and DEE (OR: 1.21, 95% CI: 0.82–1.77). No associations were detected between paternal exposure to nickel, PHA, or asbestos. Concerning maternal exposure, slightly increased odds ratios were found but with large confidence intervals due to the low number of exposed mothers. As many pesticides were used on various crops, the indicators were estimated to be moderately to highly correlated with each other (Spearman Rho: 0.30–0.88) and with the main types of crops (Spearman Rho: 0.28–0.85), except for grass/clover, which had lower pesticide applications (Spearman Rho 0.001–0.37). The inclusion of the four pesticides in the same model resulted in widened confidence intervals and modest attenuation of the associations between leukemia and fluroxipyr/bromoxynil/dioxinil (3rd tertile vs. no cultures, HR = 2.1, CI = 0.4–12, 1), tribenuron-methyl (HR = 1.9, IC = 0.5–7.1) and tebuconazole (HR = 1.9, IC = 0.5–7.1), without cultures, HR = 2.1, CI = 0.4–12.1), tribenuron-methyl (HR = 1.9, CI = 0.5–7.1) and tebuconazole (HR:1.8, 95% CI:0.5–7.0), and substantial attenuation of the association for phenmedipham (HR = 1.2, CI = 0.3–4.4). There were no associations between CNS tumors and any pesticide.

Glass and colleagues (16) conducted a study in Australia between 2003 and 2006 and the authors found no association between parental exposure to pesticides and the risk of leukemia in their children in the cohort studied. Exposure to organophosphate insecticides, organochlorine insecticides, phenoxy herbicides, other herbicides, and other pesticides was noted. A total of 416 cases and 1.361 controls participated. Professional exposure was infrequent (15%); approximately 1% of mothers were exposed before birth, and 0.1% were exposed after birth. No significant association was observed between paternal occupational pesticide exposure and an increased risk of ALL in offspring, regardless of the type of pesticide exposure (OR: 1.06; 95% CI 0.73–1.55).

The association between parental pesticide exposure and childhood leukemia is supported by most of the studies. Park et al. (17) investigated the association between prenatal pesticide exposure and ALL and AML in Californian descendants. Data were collected between 1998 and 2011 for a total of 162 leukemia patients and 9,805 controls. Elevated risks of ALL were observed with exposure to any carcinogenic pesticide (aOR: 2.83, 95% CI: 1.67–4.82), diuron (aOR: 2.38, 95% CI: 1.57–3.60), phosmet (OR: 2.10, 95% CI: 1.46–3.02), kresoxime-methyl (OR: 1.77, 95% CI: 1.14–2.75), and propanil (OR: 2.58, 95% CI: 1.44–4.63). Analyses based on chemical class revealed high risks for patients receiving 2,6-dinitroanilines (OR: 2.50, 95% CI: 1.56–3.99), anilides (OR: 2.16, 95% CI: 1.38–3.36) and ureas (OR: 2.18, 95% CI: 1.42–3.34).

In another study, Patel et al. (18), the occupational exposures of parents and the risk of childhood leukemia in their offspring were prospectively evaluated. The data were collected from 329,658 birth cohort participants in five countries (Australia, Denmark, Israel, Norway, and the United Kingdom). The risk of ALL (*n* = 129), AML (*n* = 31), and central nervous system (CNS) tumors (*n* = 158) in childhood (<15 years) was estimated using Cox proportional hazards models to determine hazard ratios (HRs) and 95% CI. Paternal exposure to pesticides and animals was associated with an increased risk of childhood AML (herbicides HR: 3.22, 95% CI: 0.97–10.68; insecticides HR: 2.86, 95% CI: 0.99–8.23; animal HR = 3.89, 95% CI: 1.18–12.90), and ALL and CNS tumors were not associated. Paternal exposure to organic dust was positively associated with AML (HR = 2.38, 95% CI: 1.12–5.07), inversely associated with ALL (HR: 0.55, 95% CI: 0.31–0.99), and not associated with CNS tumors. It was not possible to analyze maternal exposure to pesticides.

In a Chinese population, Wang et al. (19)(2019 conducted a study between 2014 and 2016 and investigating the association of maternal exposure in the prenatal period and environmental factors with the risk of ALL. A total of 345 patients were matched with controls, and interviews were conducted. An unconditional logistic regression adjusted for age, sex, region of residence, and relevant confounders was performed to generate odds ratios (ORs) and 95% confidence intervals (CIs). The risk for ALL was increased when there was prenatal maternal exposure to pesticides (OR = 1.48, 95% CI = 1.67–2.28).

In a case‒control study conducted in Costa Rica between 2001 and 2003, Hyland et al. (20) assessed whether maternal use of pesticides at home or around homes before pregnancy, after pregnancy, or during breastfeeding was associated with leukemia in offspring. Controls were selected from individuals of the same age and who lived in the same neighborhood as the patients. In total, 251 ALL patients and 577 controls were interviewed in person for data collection. The data collection times were standardized to one year before, during, and after breastfeeding. The authors adapted a logistic regression model to estimate the associations. Maternal insecticide use in the year before pregnancy, during pregnancy, and during breastfeeding were associated with increased odds of ALL among boys (aOR: 1.63; 95% CI: 1.05–2.53), 1.75 (95% CI: 1.13–2.73) and 1.75 (95% CI: 1.12–2.73), respectively. In contrast, maternal insecticide use after birth and during any period was associated with small reductions in the odds of ALL among girls aOR: 0.69 (0.39–1.23) and 0.66 (0.37–1.17), respectively. Maternal use of herbicides indoors was not associated with childhood ALL according to the combined analyses stratified by sex. Reporting pesticide spraying in places close to residences was also associated aOR: 1.43 (95% CI: 1.00–2.05), 1.41 (95% CI: 0.98–2.03) and 1.52 (95% CI: 1.11–2.09). The use of insecticides indoors was more likely to cause LLA than was the use of these chemicals alone (<2.5 times/year) OR: 1.56 (95% CI: 1.07–2.27), 1.58 (95% CI: 1.08–2.31) and 1.56 (95% CI: 1.07–2.29), respectively.

An Italian study by Ferri et al. (21) (2018) investigated the association between acute leukemia in offspring, maternal exposure during pregnancy, and paternal occupational exposure to pesticides. This case‒control study was carried out between 2010 and 2015. A total of 116 patients (101 ALL and 15 AML) and 162 controls participated in the study. Maternal exposure during pregnancy was significantly associated with leukemia incidence, domestic insecticide use and proximity of residences to application sites during the pregnancy period (OR: 1.87; 95% CI, 1.04–3.33) but was not associated with maternal pesticide use during childhood. There was no significant increase in the use of pesticides by fathers or parents during pregnancy or childhood, and concerning proximity (<100 m) to fields treated with pesticides, it was associated with leukemia (OR: 3.21; 95% CI: 1.37–7.53). During pregnancy, the association between leukemia status and maternal health was significant (OR: 1.93; 95% CI: 1.10–3.42).

In a case‒control study carried out in California between 1995 and 2008, Gunier et al. (22) (2017) reported that the risk of ALL was significantly elevated among children whose parents were exposed during the perinatal period (OR: 1.7; 95% CI: 1.2–2.5), with greater risks associated with pesticides used on nut crops (OR: 4.5; 95% CI = 0.9–23.0). The relationship with paternal perinatal pesticide exposure was stronger for children diagnosed at four years or earlier (OR: 2.3; 95% CI: 1.3–4.1). However, this effect was attenuated when less detailed exposure information was used. However, perinatal maternal exposure to pesticides has not been associated with ALL.

Bailey et al. (23) (2015) published two studies seeking to determine the association between maternal exposure to pesticides during pregnancy and leukemia risk in children. In 2015, data from studies published in different countries were analyzed to determine the associations between maternal exposure to pesticides before and during pregnancy and increased risk of developing leukemia in offspring. ALL associated with pesticide exposure shortly before conception, during pregnancy and after birth were OR: 1.39; 95% CI: 1.25–1.55) (for 2,785 patients and 3,635 controls), 1.43 (95% CI 1.32–1.54) (for 5,055 patients and 7,370 controls) and 1.36 (95% CI: 1.23–1.51) (for 4,162 patients and 5,179 controls), respectively. The prevalence of AML was 1.49 (95% CI: 1.02–2.16) (173 patients and 1,789 controls), 1.55 (95% CI: 1.21–1.99) (344 patients and 4,666 controls) and 1.08 (95% CI: 0.76–1.53) (198 patients, 2,655 controls). This positive association occurred regardless of the duration of maternal exposure and the type of pesticide (not mentioned). The results of the association are positive for both ALL and AML.

In 2014, the same author mentioned above compiled 13 case‒control studies from the International Childhood Leukemia Consortium (CILI) to assess whether maternal and paternal exposure to pesticides during pregnancy increased the risk of ALL and AML in offspring. A total of 8,236 cases and 14,850 controls were included. Through logistic regression data analysis, a significant increase in maternal exposure to pesticides during pregnancy was found for ALL (OR: 1.01; 95% CI: 0.78–1.30), and for paternal exposure, it was 1.20 (95% CI: 1.06–1.38). In AML patients, the OR for maternal exposure during pregnancy was 1.94 (95% CI: 1.19–3.18), and for paternal exposure, it was 0.91 (95% CI: 0.66–1.24) based on data from 1,329 cases and 12,141 control mothers and from 1,231 cases and 11,383 control fathers. Another interesting finding was in children aged five years or older or with T-ALL, whose risk increased according to paternal exposure at the conception stage (24).

In China, between 2009 and 2010, Shi et al. (25) explored the relationship between parental exposure to various chemicals and the risk of childhood acute leukemia in their offspring. This was an exploratory case‒control study involving 201 patients with acute leukemia carried out through interviews with mothers. Among the chemicals analyzed whose mothers were exposed were gasoline, diesel oil, paints, chemical fertilizers, pesticides, herbicides, and insecticides three months before pregnancy until the end of pregnancy (OR: 2. 9; 95% CI: 1.1–7.8), paternal exposure to insecticides (OR: 10.1; 95% CI: 1.2–82.9) and chemical fertilizers (OR: 9.5; 95% CI: 1.1–79. 6). In the three months prior to pregnancy, maternal work experience in agriculture and forestry before pregnancy (OR: 8.4; 95% CI: 1.4–50.2) and in spinning, leather processing, vehicle decoration and repair before pregnancy (OR: 3.0; 95% CI: 1–7.9), during pregnancy (OR: 3.2, 95% CI: 1.1–9.6), and during paternal work in agriculture and forestry (OR: 9.6; 95% CI: 2.1–44.8) and spinning, leather processing, vehicle decoration and repair (OR: 2.3; 95% CI: 1.1 −5.0). Parental exposure to these evaluated products, including pesticides, may increase children's leukemia risk.

A Brazilian study was conducted by Ferreira et al. (26) (2013) from 1999–2007. A total of 252 patients and 423 controls were recruited, and their data were collected before, during, and after pregnancy. A positive association was found concerning acute lymphoid (aOR: 2.10; 95% CI: 1.14–3.86) and myeloid (aOR: 5.01; 95% CI: 1.97–12.7) leukemia in children aged 0–11 months and with ALL (aOR: 1.88; 95% CI: 1.05–5.23) at 12–23 months of age. With respect to reported maternal exposure to permethrin, higher risk estimates were found for children aged 0–11 months (aOR: 2.47; 95% CI: 1.17–5.25 for ALL; and aOR: 7.28; 95% CI: 2.60–20.38 for AML). Maternal exposure to pesticides related to agricultural activities had an aOR of 5.25 (95% CI: 1.83, 15.08) for ALL and an aOR of 7.56 (95% CI: 1.83–31.23) for the AML.

Ruth et al. (27) conducted a study examining the relationship between maternal and paternal household pesticide exposure during pregnancy and the risk of childhood ALL. The study included 1,810 cases of children aged 15 years or younger, identified through Children's Cancer Group institutions between 1989 and 1993, and matched by age and sex to 1,951 controls. Data on household pesticide use during pregnancy and the month prior were collected via telephone interviews. A comparison group was established, consisting of participants reporting no parental exposure to 10 pesticide classes. When restricting the comparison to the unexposed group, adjusted odds ratios increased by 15%–49%. Notably, maternal exposure to termite insecticides was associated with the highest ALL risk (aOR: 4.21; 95% CI: 2.00–8.88).

## 4 Discussion

In this pooled analysis, we reviewed 14 studies that linked parental exposure to pesticides and the risk of childhood leukemia in offspring. Although the current literature does not provide robust evidence regarding the causes of leukemia, 12 of these studies indicated a positive association.

The study by Patel et al. (28) was the first to prospectively examine the association between farming areas, the number of animals near homes, and the risk of childhood leukemia and CNS tumors. The use of pesticides was evaluated, classified as eight herbicides, and one fungicide was applied in the evaluated crop areas. The findings of this study demonstrated an increased risk of childhood leukemia in children of mothers with higher tertiles of application of the herbicides fluroxipyr/bromoxynil/dioxinil, phenmedifam, and tribonuron-methyl and the fungicide tebuconazole in cultivation areas close to their homes during pregnancy. Proximity to agricultural areas increases the concentration of pesticides in the air and consequently increases their precipitation. In these regions, pregnant women have higher urinary metabolite levels than women who do not live in agricultural areas (29, 30). Furthermore, the fungicide tebuconazole is widely used in agricultural practices, especially for cereal crops, and its potential cardiotoxicity has already been noted (31).

Considering that many pesticides, including glyphosate and pendimethalin, have significant residential applications, a limitation of this study was the need for additional information on exposure, as the use of pesticides in homes and gardens was not evaluated; rather, only cultivated areas were included. On the other hand, information on the names of pesticides is lacking in other studies, even though the correlated use of pesticides also did not make it possible to evaluate them individually. However, the use of a single period (during pregnancy) and the use of individual metrics are positive factors. Another limiting factor was the small number of exposed patients, which resulted in a small sample size. On the other hand, an increased risk of childhood leukemia was associated with greater use of two of the herbicides, which were applied to most hectares of spring cereals and had more limited use in winter cereals and seed crops, as was an estimated combination of bromoxynil, ioxynil, and fluroxypyr, which were used on winter and spring cereals and corn.

Glyphosate is the most widely used herbicide in the world, and environmental or occupational exposure can cause epigenetic alterations in individuals; for example, it alters global methylation in various types of cells and organisms and is responsible for the methylation of individual promoter genes. It is understood that these mechanisms are potentially involved in oncogenesis; however, the role of glyphosate in the development of cancer and its toxicity mechanisms should be further studied (32).

Like previous studies that aimed to assess exposure to pesticides during pregnancy, Park et al. (17) extended their study in a very positive way by identifying specific exposures to pesticides and by analyzing leukemia in ALL and AML patients separately; however, most of those studies were limited to analyzing leukemia in general. This narrowing down is interesting since the subtypes of leukemia have different characteristics, and each pesticide has its own mechanism of action. Another notable aspect of this study was the use of an advanced tool, the Geographic Information System (GIS), which they developed to integrate land use data with pesticide records. This tool was applied to all rural areas in California that use agricultural pesticides at radii of 500 m, 2000m and 4,000 m. The authors suggested that in rural areas of California, exposure to 2,6-dinitroanilines, anilides, and ureas, specifically diuron, phosmet, cresoxim-methyl, and propanil, increases the chances of ALL among children exposed during pregnancy, as they are related to the proximity of pregnant mothers to agricultural areas. Additionally, exposure to metam sodium and paraquat dichloride may increase the chances of AML.

Despite this association, the study faced recall bias regarding parental pesticide exposure, as the results were based on ecological or case‒control studies. Furthermore, the author showed that paraquat is an agent that causes oxidative stress and damage to mitochondrial DNA, supporting the mechanism that may be linked to the increased risk of developing AML (33). In addition, exposure to metam sodium and paraquat dichloride can increase the risk of AML, and this second active ingredient is also associated with neurotoxicity, contributing to the development of other diseases, such as Parkinson's disease (34). This is the only study in California in which exposure was evaluated using specific information about housing and the duration of pregnancy. Despite this, there were also limitations, as in any study. Live birth bias was present since miscarriages that occurred due to high exposure were not accounted for, and household information was only available at the time of birth. There was also a lack of information on occupational and domestic exposure, which may underestimate the results since there are studies in this area (24).

As already mentioned, Patel et al. (18) performed a pooled analysis of five international cohorts (Australia, Denmark, Israel, Norway, and the United Kingdom) of children up to 15 years of age. For the first time, they demonstrated an increased risk of AML related to paternal exposure to pesticides. An increased risk of AML has previously been reported for maternal occupational exposure to pesticides and other occupational exposures. A positive factor of this analysis was the study design itself, as it was a prospective analysis, avoiding selection and recall bias, and could be used to examine possible confounding factors, such as smoking, birth weight, and breastfeeding. Various occupational exposures were also assessed using ALOHA and JEM, that is, standardized occupational codes, which reduced the likelihood of memory bias related to questioning specific occupational exposures. Unlike other authors, in this study, the authors also assessed parental exposure to farm animals and organic dust. With regard to animals, according to the authors, the risk is due to exposure to zoonotic viruses and microbes, although the number of exposures was low. However, limiting factors in this study were based on a risk estimate based on a relatively small sample of exposed individuals due to a low prevalence of exposure to pesticides and animals, preventing the assessment of the risk associated with maternal exposure in these studied cohorts. Similarly, another limiting factor was the heterogeneity of occupational exposure categories, as they were composed of many different pesticides and types of organic dust, making it impossible to measure the toxicity and potential biological effects of these substances individually. Moreover, information about the duration and detailed history of occupational exposure also limited the findings of this study. Despite these limitations, this study was the first to find a positive association between paternal occupational exposure and an increased risk of developing AML in children.

To investigate the association between prenatal maternal exposure to various environmental factors and the risk of ALL in childhood and possible interactions in the Chinese population, Wang and colleagues (19) (2019) noted a positive association between prenatal maternal exposure to various environmental factors and an increased risk of ALL in children, despite not revealing the interaction of these factors with each other. This study had a relatively large sample size and evaluated several covariates, including a family history of leukemia and other types of cancer in the child, child exposure to pesticides, age of parents at birth, parental education level, and excessive alcohol consumption by the father. However, the limiting factors observed were the lack of exposure characterization, as the results were heterogeneous, as the reported exposures were domestic or occupational but did not define the type of pesticide used by the parents; selection bias is also limiting, as controls were not randomly selected from the population but from a hospital sample. Among the factors assessed in the present study, home interior renovation, a very common practice in China, was found to be an increased risk factor along with exposure to pesticides. Despite being a regional practice, people are exposed to environmental pollution during pregnancy, which can occur in other places or regions, especially for women who become pregnant sequentially. As a result of this exposure, it was possible to note a risk associated with childhood ALL. However, in this case, the participants were exposed to paints, paving and new furniture, and similar results were observed with respect to exposure to chemicals such as benzene (35). With regard to pesticides, the analyses were limited to domestic exposures only due to the low number of occupationally exposed mothers. Even so, an increased risk for ALL was observed, in agreement with the findings of similar studies (36, 37).

Similarly, another case‒control analysis conducted in Costa Rica by Hyland et al. (20) demonstrated that maternal use of insecticides indoors and the spraying of pesticides on farms or businesses near the home during pregnancy or while breastfeeding were associated with an increased risk of leukemia among boys; moreover, these authors found a greater chance of ALL in children whose mothers reported handling these insecticides more frequently during pregnancy or breastfeeding.

A recent study revealed statistically significant results for pediatric leukemia after the expansion of agriculture and, consequently, the increased use of pesticides, raising questions about the process of carcinogenesis due to these exposures (38). Another relevant fact was the possibility of residual confusion attributed to parental occupational exposure to pesticides, as Costa Rica has one of the highest rates of agricultural pesticide use in the world, directly impacting the assessment of different routes of exposure, especially in children who reside in agricultural areas. Furthermore, a study was conducted in the first country in Central America to examine the associations between the domestic use of pesticides and childhood leukemia, and the authors also noted a difference between the sexes of children concerning this exposure a positive result for males (20).

According to Ferri et al. (21), the factors associated with leukemia are maternal use of pesticides during the gestation period, living in places close (<100 m) to fields treated with these compounds, and paternal occupation in the military. Parental occupational exposure was not associated with this study; however, other studies showed contrary results (16, 24, 39). Although the sample size was small, perhaps because of the contradictory results, these findings provide a warning about the relevance of the topic. Although the domestic use of pesticides is often considered harmless, studies have shown otherwise (37, 40). Another study showed the association between living in places close to agricultural fields and the risk of developing leukemia in babies during the gestational period (41). Additionally, the authors suggest paternal occupation in the armed forces as a risk factor for the development of leukemia because of contact with many people and the chances of contracting viral infections, one of the pathogenicity mechanisms of malignant hematological diseases (42).

Finally, the possible association between the CYP2D6*4 polymorphism and the etiology of childhood leukemia was also demonstrated; however, these findings are conflicting and have already been discussed in the literature (43). The present study presented unstable estimates due to low cases and estimates with a type 1 alpha error of 0.05%, which was significant but not very robust. As a result, it was also impossible to analyze the different leukemias or pesticides used individually. The mothers' participation during the interviews was positive, representing 86% of the participants. The factors associated with pesticide exposure had an OR: 1.87 and the expected OR: 2.0. A worrying factor is the memory bias during the interviews, which may have caused errors in their classification since the categories are broad, and it is not possible to identify specific products. Therefore, these findings do not invalidate the importance of the subject discussed here or exhaust it since each study brings about a reality and truth inferred in its results.

The California Childhood Leukemia Study (CCLS), in another case‒control study conducted in 35 counties in the state (22) used a methodology developed to specify and improve exposure assessment. These are closed and branched questions based on tasks and a calendar with significant life events evaluating exposure in critical periods. The CCLS is a large case‒control study based on the local population. They ensured that as much information was recorded from one year before the child was born until the child was three years old or was diagnosed regarding the frequency, intensity, and duration of exposure. In addition, detailed information was collected from parents regarding their jobs since the age of 18, mainly concerning the use of pesticides and the type of role they performed. Paternal perinatal exposure was associated with a 70% increased risk of ALL, while maternal exposure was not associated with this risk. This result contrasts with the study by Glass et al. (16), which will be discussed below but finds no association between paternal exposure and an increased risk of leukemia. The low prevalence of maternal exposure during pregnancy weakened the study. These results were similar to those of the International Childhood Leukemia Consortium (CILI), which revealed an increased risk of infection in exposed parents (OR: 1.2, 95% CI: 1.1–1.4); however, another study revealed a more significant risk when both parents were exposed (44). It was impossible to evaluate the pre- and postnatal periods as essential exposure windows separately. Individual pesticides were also not associated, and grouping may have attenuated the estimates. The use of specific modules for comparison with estimated exposure is a positive point, and the study population was superior to that of previous studies with similar comparison methods.

For Bailey and colleagues (23) all exposure to pesticides, whether before conception, during pregnancy, or after birth, presents an increased risk of leukemia, and this is independent of the period or type of pesticide. The father's exposure can cause damage to germinative cells. Moreover, when pregnant, the mother can expose the baby, a fact already demonstrated when analyzing umbilical cord blood and the baby's meconium (45).

Pesticides have different characteristics and toxicity properties. Exposure patterns were similar in the periods analyzed, making it impossible to assess critical exposure periods. The type of pesticide used remained the same, possibly due to simultaneous exposure to more than one category, making it impossible to include all classes in a logistic regression model. The coarse and general measurement of these data and the lack of pesticide specificity are areas for improvement in the study. Memory bias must also be considered since the data are from self-assessments, which can significantly affect the results. Ideally, there would be biomarkers that could be measured and quantified *in utero*, but this is impossible. The strengths of this study include the use of many samples and access to original data and other information, such as immunophenotype and cytogenetic data, which contributes to more robust work.

**Figure 2:**
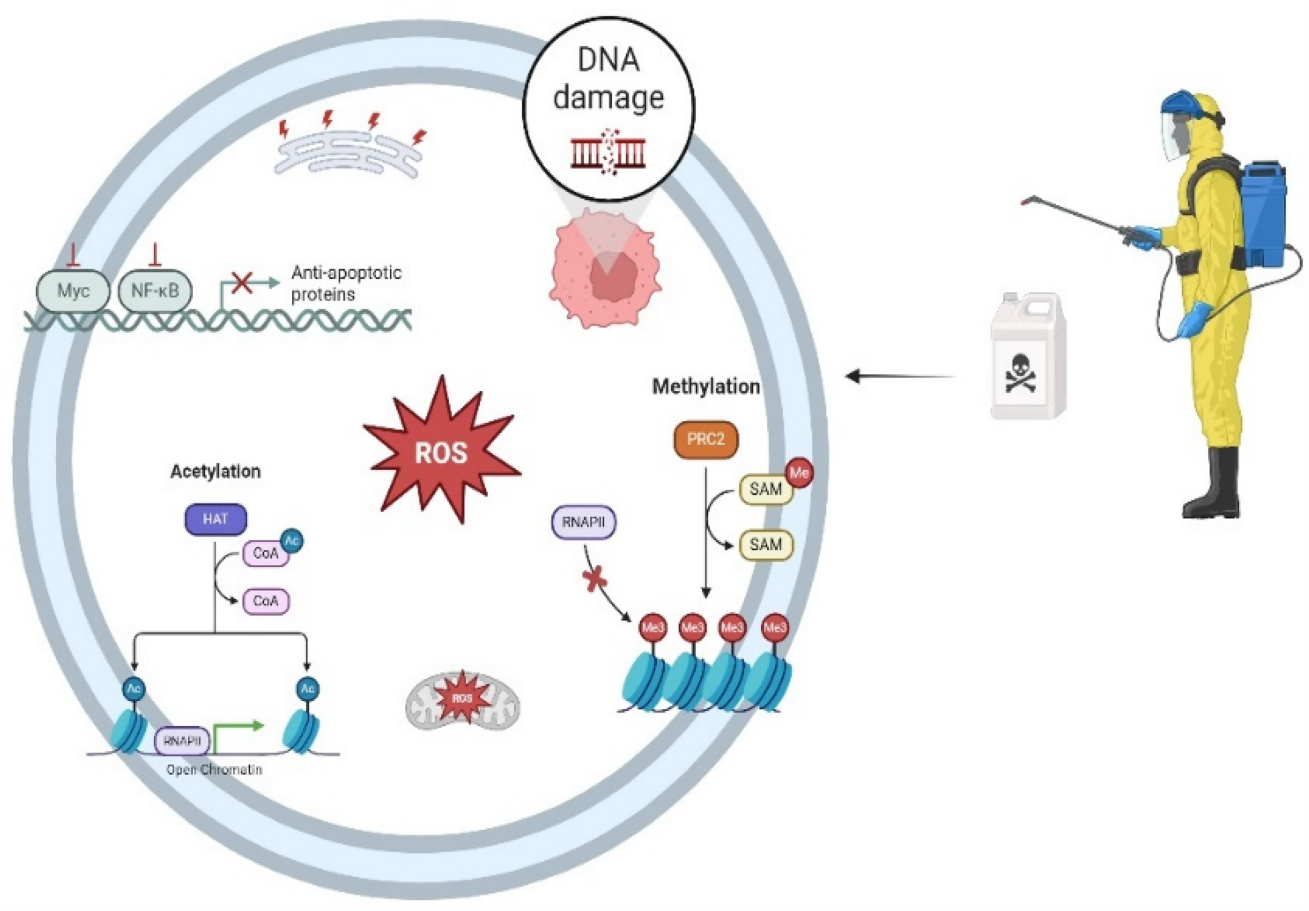
Multiple mechanisms connecting parental pesticide exposure to DNA damage. RNAPII, RNA polymerase II; PRC2, polycomb repressive complex 2; SAM: S-adenosyl methionine; HAT: histone acetyltransferase; COA: coenzyme a; ROS: reactive oxygen species; Myc: Oncogene that acts as a universal amplifier transcription fator; NF-Κb: nuclear factor kappa-light-chain-enhancer of activated B cells; DNA: deoxyribonucleic acid. Created in https://BioRender.com.

Continuing with Bailey et al. (24), the aim was to investigate whether parental exposure to pesticides in the prenatal period could increase the risk of leukemia in their children and whether this relationship changes according to the immunophenotype of leukemia. Furthermore, the results differ between subtypes for exposure from both parents. Maternal exposure increases the risk for AML, while paternal exposure is more remarkable for ALL. Another study highlighted the risk factors for maternal exposure and AML in the home environment (46). Thus, oncogenesis may be linked to parental exposure during the preconception and intrauterine periods, and potential mechanisms of DNA damage include DNA methylation, acetylation, ROS generation, and the expression of anti-apoptotic proteins, among others (Figure 2).

Ferreira et al. (26) extended their analyses from another previously published study with the same objective of evaluating the risk of maternal exposure to leukemia in offspring. The results showed that mothers exposed to pesticides three months before conception were twice as likely to be diagnosed with ALL in their children's first year than unexposed mothers were. These data are consistent with the fact that, because these children developed cancer so early, the carcinogenesis process may have begun in the mother's womb, corroborating the hypothesis proposed by Greaves et al. (42) that chromosomal translocations begin in the prenatal period. Among the limitations, the hospital-based study design (case‒control) could introduce selection bias, and cases and controls with similar origins could induce an overlap in exposure. The duration of exposure was also not evaluated, and it was impossible to estimate the cumulative exposure. Another area for improvement was the sample size, especially concerning LMA, which resulted in inaccurate estimates. Of the strengths, the study was based on children under two years of age; it also provided information on the type of exposure, exposure to individual products, mainly pyrethroids, and data on leukemia subgroups, improving the understanding of the subject.

Glass et al. (16), aimed to evaluate the association between parental occupational exposure and leukemia in children but found no significant relationship. Although an excellent method was used to assess exposure, which minimizes memory bias when compared to self-assessment methods, this result is contrary to the literature and was justified by the low prevalence of exposure among women and the low participation of controls, making the study's conclusions difficult. In addition, the type of pesticide used at work and at home may be different, and the length of exposure in these places also differs. Notably, personal protective equipment may have been used at work, which was not the case at home. Despite this, it is important to point out that exposure to pesticides can also occur through routes other than occupational or conventional domestic use. The presence of pesticide residues in both food and water has already been found in quantities beyond those permitted by Brazilian legislation or even pesticides banned for use in the country (6, 47).

Finally, the study of Rith and cols (27) carried out research exploring the connection between parental pesticide use at home during pregnancy and the risk of childhood ALL. The study analyzed 1,810 cases of children aged 15 or younger, identified through Children's Cancer Group institutions between 1989 and 1993, and matched by age and sex with 1,951 controls. Information on pesticide use in the household during pregnancy and the preceding month was gathered through telephone interviews. To refine the analysis, a reference group (named as NPE10) was created, consisting of individuals who reported no parental exposure to 10 specific pesticide types. When limiting comparisons to the NPE10 group, adjusted odds ratios increased by 15%–49%. Among the exposures assessed, maternal contact with termite insecticides showed the highest associated risk (adjusted odds ratio: 4.21; 95% confidence interval: 2.00–8.88). This study benefits from a large sample size, age- and sex-matched controls, and a refined comparison group (NPE10), focusing on maternal and paternal pesticide exposure during pregnancy. However, it has limitations, including reliance on self-reported data, potential recall bias, and lack of direct biological exposure measures.

Some of the included studies identified a strong link between parental pesticide exposure and leukemia. Several factors may explain these discrepancies, including variations in study design, differences in sample sizes, geographic location, genetic backgrounds, lifestyle influences, and exposure to additional environmental carcinogens. Another key issue is the variability in exposure assessment methods—some studies rely on self-reported data, which can introduce subjectivity and potential bias. Furthermore, inconsistencies in the timing and duration of exposure, the specific types of pesticides examined, exposure to mixtures, and the lack of detailed pesticide classification can impact findings. The measurement of pesticide residues in biological samples, such as blood and urine from both parents and offspring, also varies across studies, potentially affecting comparability. Lastly, methodological constraints, such as small sample sizes or insufficient control of confounding factors, may contribute to conflicting outcomes. Despite these inconsistencies, the relevance of these studies remains significant, emphasizing the complexity of investigating environmental risk factors for childhood leukemia and the urgent need for more standardized, high-quality research.

## 5 Conclusions

This review found that 86% of the studies analyzed reported a positive association between occupational pesticide exposure and the development of leukemia. However, limitations in the study design, such as heterogeneity and the number of participants from the different populations studied, make it difficult to accurately characterize occupational exposure, and prevent the identification of specific assets responsible for these associations. Therefore, it is known that multiple factors contribute to the pathogenesis of leukemia, and parental exposure to pesticides may be a significant risk factor, and the implementation of public health policies aimed at reducing parental exposure to environmental toxicants could potentially contribute to reducing the incidence of several malignancies, including leukemia. However, research is needed to strengthen the evidence base, particularly regarding the mechanisms of leukemogenesis and the role of environmental exposures as contributing risk factors.
